# Kinetico-Mechanistic Study of the Chemical Redox Cycling of Cubic PBA {M^+^} ⊂ (Co^III^_4_/Fe^II^_4_) Structures

**DOI:** 10.1021/acs.inorgchem.5c05805

**Published:** 2026-05-08

**Authors:** Miguel A. Gonzálvez, Montserrat Ferrer, Manuel Martínez

**Affiliations:** † CNRS/Université de Toulouse, Laboratoire Hétérochimie Fondamentale et Appliquée (LHFA, UMR 5069), 118 Route de Narbonne, Toulouse, Cedex 09 31062, France; ‡ Secció de Química Inorgànica, Departament de Química Inorgànica i Orgànica, 16724 Universitat de Barcelona, Martí i Franquès 1-11, Barcelona 08028, Spain; § Institute of Nanoscience and Nanotechnology (IN2UB), Universitat de Barcelona, Barcelona 08028, Spain

## Abstract

We report a kinetic and electrochemical study of the four-electron redox processes (Fe^II^/Fe^III^) in cyanido-bridged cubic structures {M^+^} ⊂ (Co^III^_4_/Fe^II^_4_), (M = {K^+^}, {Na­(OH_2_)^+^}). Oxidation with S_2_O_8_^2–^ is kinetically inhibited in neutral/borate media and requires >10^3^-fold oxidant; at pH = 0 the four Fe^II^ → Fe^III^ steps become resolvable. Time-resolved UV–vis reveal four sequential oxidations whose second-order rate constants decrease by 1–2 orders of magnitude per step; Eyring analysis shows modest Δ*H* ^⧧^ values and increasingly negative Δ*S* ^⧧^ with advancing oxidation steps. The {Na­(OH_2_)^+^}-encapsulated cube oxidizes over a narrow time window and, on full oxidation, ejects its encapsulated cation, whereas the symmetrical {K^+^} cube displays more widely spaced kinetics between steps and retention of the encapsulated cation. Partial reduction of the oxidized species at alkaline pH proceeds through three consecutive steps but is limited by the insolubility of fully oxidized material. Heterogeneous oxidation by cyclic voltammetry indicates that low external [Na^+^] also promotes expulsion of {Na­(OH_2_)^+^}, while the larger {K^+^} cation remains encapsulated even at high [Na^+^], as indicated by the lack of changes in the electrochemical reversibility.

## Graphical abstract

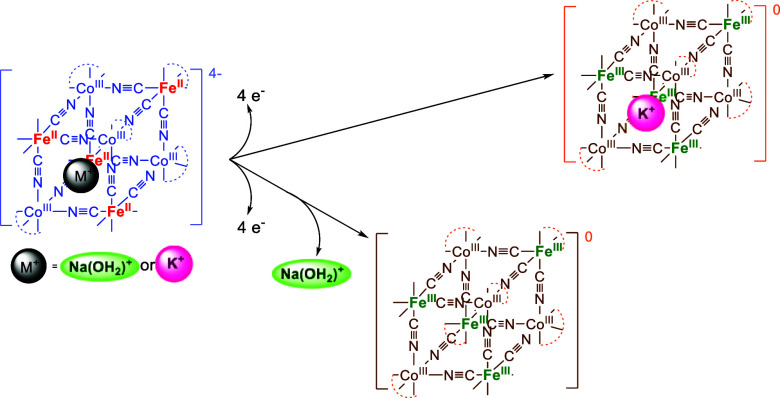

## Introduction

Mixed-valence compounds have long attracted scientific interest due to their physical and chemical properties arising from electron delocalization between metallic centers.^1,2^ Prussian Blue (PB) and its analogues (PBAs) are coordination frameworks of transition metals linked through cyanide bridges (–C≡N–); while in the case of PB linkage is between low-spin Fe^II^ and high-spin Fe^III^ sites, thus producing mixed-valence species, for PBAs the existence of distinct metal sites lead to species that, even sharing some of their properties, are not mixed-valence.^3,4^ In both cases, electron delocalization enables a metal-to-metal charge transfer (MMCT), which has an influence on some of the characteristic chemico-physical properties of PBAs, such as color tunability or redox activity and can be modulated by external stimuli.^5^ On the other hand, the contribution of charge tranfer to magnetic interactions has been proposed only in certain cases.^6,7^

For PBAs, changes of transition metals and/or ancillary ligands create a delicate interplay between properties and functions, directly affecting electronic and lattice configuration, charge delocalization, etc.^8,9^ Among these, Co–Fe PBAs generally show metal–metal electron transfer coupled to a spin transition on the cobalt (sometimes called ETCST),^10–12^ which are sought for phenomena in molecular electronic, and photomagnetism material research.^13,14^ Another example of how PBAs show interesting structural-function properties is found in some open-framework structure PBAs used for cathode materials, where the reversible caging and expelling of guest cations such as Li^+^, K^+^ and Na^+^ take place, making them attractive for energy storage applications.^15–17^ In these systems, redox processes at the metal sites govern charge storage, with the MMCT mechanism facilitating charge compensation and electron transport within the framework. Co–Fe compounds of the type A_2_Co_4_[Fe­(CN)_6_]_3_._3_·*n*H_2_O (A^+^ = Na^+^, Rb^+^, Cs^+^), have shown that alkali cations dictate the electronic ground state; Na^+^ structures undergoing thermally activated Co^II^ (high-spin)–Fe^III^ → Co^III^ (low-spin)–Fe^II^ electron (MMET) transfer upon cooling, whereas Rb_2_CoFe and Cs_2_CoFe already adopt the Co^III^ (low-spin)–Fe^II^ configuration at room temperature.^18^

Due to the fact that PBAs exist as extended 3D solid-state frameworks, which are inherently complex, the study of the fundamental electron transfer processes that dictate their macroscopic properties becomes very demanding;^19^ stability, solubility, and maintenance of the structural integrity are often common challenges.^20^ Consequently, it is important to gain a deeper understanding of how structural factors influence electronic coupling and redox behavior to proceed to a rational design of functional material and harness their unique characteristics. Recent efforts to design discrete molecular models of PBAs of lower dimensionality have evolved significantly over the years, enabling a fine synthetic control over physicochemical properties that these models offer.^21^ In this respect, a recent report on a series of cyanide-bridged (Fe_4_Co_4_) cubic structures indicates that the variation of the external counteranion (BF_4_^–^, ClO_4_^–^, PF_6_^–^, AsF_6_^–^, SbF_6_^–^) strongly modulates the metal-to-metal electron transfer.^21^ Computational and experimental analyses have also revealed that anion-dependent changes in intermolecular π···π interactions between dipyridoquinoxaline ligands distort the CoN_6_ octahedra and alter the cobalt ligand field, thereby controlling MMET behavior.^22^

Relative to the context of this study, the effect of encapsulated counterions within the molecule has been shown to noticeably influence redox properties. In particular, the presence of an encapsulated sodium cation within (Fe_4_Ni_4_) cubic structures has been shown to induce a positive shift in the Fe^III^ reduction potentials, thus permitting the isolation of the mixed-valence Na^+^ ⊂ (Fe^III^_2_Fe^II^_2_Ni^II^_4_) species;^23^ while Cs^+^ produces much stronger electronic communication between the Fe centers in analogous (Fe_4_Ni_4_) cubes.^24^ Comparative redox studies revealed that the replacement of encapsulated K^+^ by Cs^+^ in K^+^ ⊂ (Co_8_), K^+^ ⊂ (Rh_4_Co_4_) species facilitates the oxidation processes.^25^ Furthermore, for some (Fe_4_Co_4_) compounds the Cs^+^ ⊂ (Fe_4_Co_4_) structure features a reduced separation between the successive cyclic voltammetry signals of the iron ions compared with K^+^ ⊂ (Fe_4_Co_4_) counterparts (*i. e.* weaker electronic communication) and additional Co-centered redox voltammetric signals.^26^ In the same line, the systematic change of the encapsulated cation (K^+^, Rb^+^, Cs^+^) reveals that the smallest K^+^ cation produces larger geometrical changes associated with the redox state variations in (Fe_4_Co_4_) cages studied.^27^ Interestingly, all the studies detailed above have been carried out in organic solvents, given the insolubility of the described molecular PBAs in aqueous media, and studies typically focus on the heaviest alkaline cations (K^+^, Rb^+^ and especially Cs^+^), while the lighter Li^+^ and Na^+^cations are rarely investigated.

Our group has been involved for some time in the preparation of a broad family of class II cyanido-bridged Co^III^/Fe^II^ complexes exhibiting some metal–metal electronic delocalization of the type indicated in Scheme 1 with different penta-, tetra-, and triaza cobalt encapsulating ligands.^28–36^ These species proved ideal for investigating the tuning of MMCT and redox behavior (Figure 1).

**Scheme 1:**
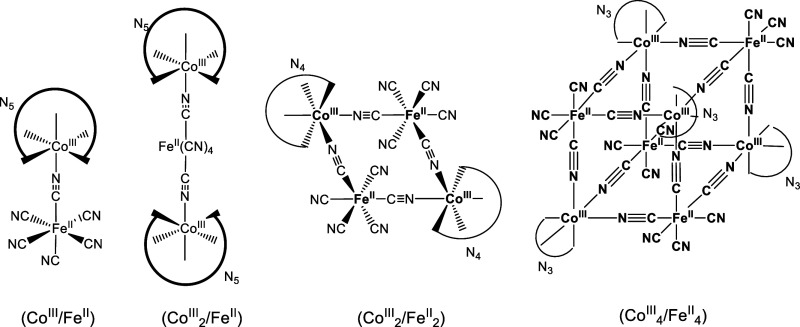


**Figure 1:**
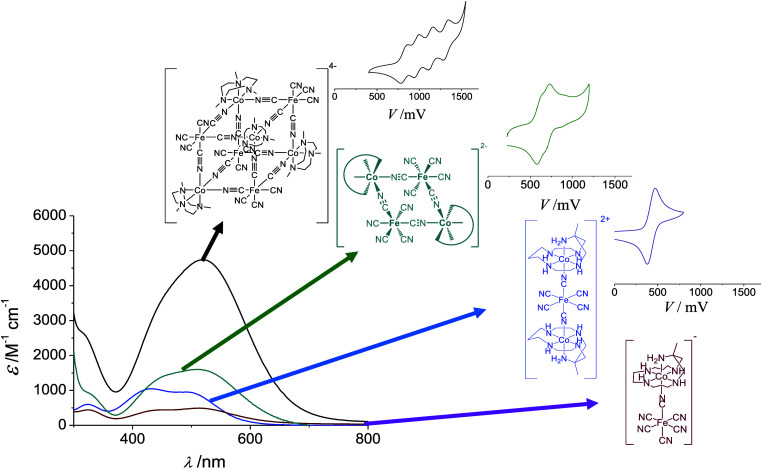
Comparison of the position and intensity of the MMCT band of the family of compounds referred in this work and selected cyclic voltammograms in the iron region.

In this context, oxidation of the Fe^II^ centers with peroxodisulfate, and their back reduction in alkaline medium have been established for the (Co^III^/Fe^II^) and (Co^III^_2_/Fe^II^_2_) compounds (Scheme 1), and these systems have been found to behave catalytically in the production of hydrogen peroxide.^37–39^ For the (Co^III^_4_/Fe^II^_4_) species described (hereafter referred to as cube), [{Co^III^(Me_3_-tacn)}_4_{Fe^II^(CN)_6_}_4_]^4–^,^40^ a complex behavior involving alkaline cationic units ({Li­(OH_2_)^+^}, {Na­(OH_2_)^+^}, {K^+^}, {Rb^+^} and {Cs^+^}) encapsulated within the cubic cavity has been thoroughly studied.^35,40^ These studies revealed that the smaller {Li­(OH_2_)^+^} and {Na­(OH_2_)^+^} (Figure 2) can exchange with external larger cations, whereas the larger {K^+^}, {Rb^+^} and {Cs^+^} remain encapsulated during the outer-sphere redox/fast substitution/inner-sphere redox mechanism operating for the self-assembly process.^41^ Furthermore, electrochemical experiments showed that for the cubic (Co^III^_4_/Fe^II^_4_) systems containing a symmetrical alkaline cationic unit within the cavity, the cyclic voltammograms are electrochemically reversible, with a clear separation of four Fe^II^/Fe^III^ oxidation events (see Figure 1). In contrast, when the cavity hosts an asymmetrical unit (specifically, {Li­(OH_2_)^+^} or {Na­(OH_2_)^+^}) the electrochemical profile loses reversibility and becomes more complex.^40^

**Figure 2:**
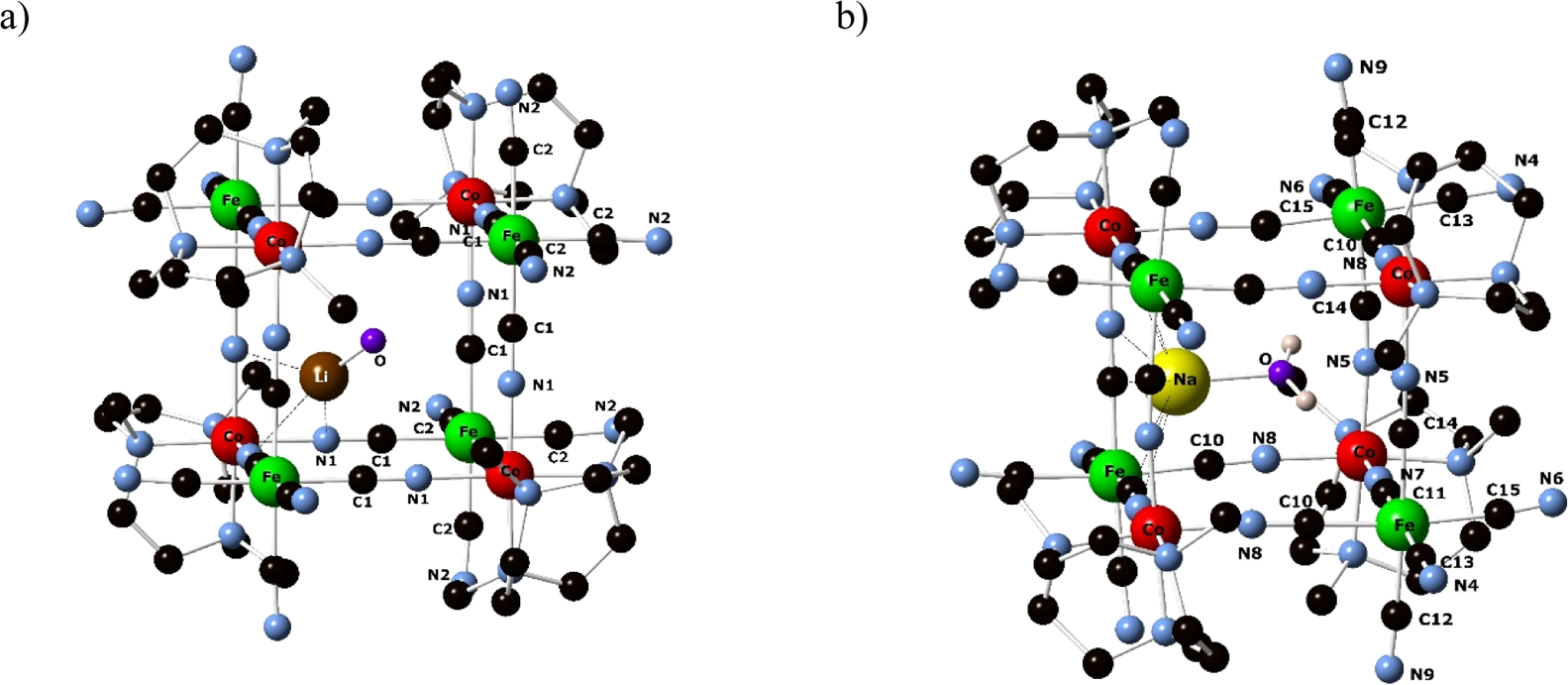
Crystallographic structures for the anionic compounds: (a)­{Li­(OH_2_)^+^} ⊂ (Co^III^_4_/Fe^II^_4_); (b) {Na­(OH_2_)^+^} ⊂ (Co^III^_4_/Fe^II^_4_). Reproduced From ref 40 Copyright 2021 American Chemical Society.

In this work, we investigate the redox processes involving the {Na­(OH_2_)^+^} ⊂ (Co^III^_4_/Fe^II^_4_) and {K^+^} ⊂ (Co^III^_4_/Fe^II^_4_) cubes and compare their behavior with the previously studied (Co^III^/Fe^II^) and (Co^III^_2_/Fe^II^_2_) systems shown in Scheme 1. The study was expected to feature a rather complex behavior given the possible occurrence of up to four reduction/oxidation processes, as well as the anticipated insolubility of the fully oxidized (Co^III^_4_/Fe^III^_4_) species. As mentioned before, studies on molecular PBAs have demonstrated that redox behavior can depend strongly on the nature of the encapsulated cationic species, a factor that plays a key role in aqueous redox catalysis and therefore warrants further investigation.^42–46^ Furthermore, while the maintenance of the portal size of the cubic complexes upon oxidation (Fe^II/III^–CN bond length being equivalent) could not be held responsible for the encapsulated cationic units to escape, the decrease of negative charge upon oxidation (as well as the protonation of the complex in some of the experiments conducted) of the cage should produce weaker electrostatic interactions, thus enabling the expulsion of the cationic units from the cube.^20,35,36,40,47^ Building on these observations, the redox processes of study here are investigated via chemical electrochemical approaches to provide valuable molecular-level insights into PBA design, with implications into their applications (energy storage, magnetic materials, stimuli-responsive systems such as photomagnetic and electrochromic devices, etc.).^17,48^

## Results and Discusion

### Chemical Oxidation

The chemical oxidation of the Fe^II^ centers of the structures prepared was investigated using aqueous solutions of Na_2_S_2_O_8_, following procedures previously applied to lower-nuclearity species of the same family.^37–39^ Interestingly, under the standard experimental conditions employed for this type of studies (oxidant concentration in a 50–200 fold excess),^37^ practically no oxidation was observed (Figure S1). Only when very large excesses of oxidant were used (>1000-fold) was a clear appearance of the characteristic Fe^III^ hexacyanido CT band at 420 nm. It is evident that although oxidation occurs, as expected from the electrochemical data of the cube, the reduction process of the (Co^III^_4_/Fe^III^_4_) oxidized species^39^ in water to produce H_2_O_2_ must be fast enough to avoid the buildup of this species, except when the oxidation process is dramatically accelerated by huge excesses of oxidant.^36,39^ In this respect, it is also clear that the outer-sphere oxidation process of the (Co^III^_4_/Fe^II^_4_) cube by the S_2_O_8_^2–^ anion must show a rather low outer-sphere pre-equilibrium constant (leading to a rate law of the standard form *k*_obs_ = *k*_et_ × *K*_OS_ × [S_2_O_8_^2–^]), similar to that of the dinuclear Co^III^/Fe^II^ complexes of the same family.^37^ This rate law accounts for the observed enormous increase in the reaction rate with the oxidant concentration. However, this behavior contrasts with that of the square (Co^III^_2_/Fe^II^_2_) structures, for which a limiting rate law (that is, *k*_obs_ = *k*_et_ × *K*_OS_ × [S_2_O_8_^2–^]/(1 + *K*_OS_ × [S_2_O_8_^2–^]) (eq S1), is observed. This implies that the reaction rate no longer increases linearly with [S_2_O_8_^2–^], even at very large concentrations, and remains constant at *k*_et_, due to a strong outer-sphere pre-equilibrium saturation kinetics, (being 1+ *K*_OS_ × [S_2_O_8_^2–^] ≈ *K*_OS_ × [S_2_O_8_^2–^]).^39^ The increase by two negative units of the charge of the species to be oxidated (from square to cubic) leads to a less favorable charge combination in the outer sphere complexation, which can easily explain the decrease of *K*_OS_.

Given these facts, and considering that the reduction of di- and tetranuclear Co^III^/Fe^III^ species by water has been shown to accelerate at high pH,^38^ the oxidation studies were subsequently conducted at pH 0. Although at this pH the {K^+^} ⊂ (Co^III^_4_/Fe^II^_4_) cube is expected to exist as a triprotonated highly stable species,^36^ its oxidation is neatly observed using the standard 50–500-fold excess of oxidant (Figure 3a). Even more, the absence of isosbestic points indicates that the process is nonstatistical, and that each Fe^II^ center reacts at a different rate, as far as the oxidation processes are concerned. This is in agreement with the independent behavior observed in the electrochemical experiments (see below) and previous data on the square (Co^III^_2_/Fe^II^_2_) structure.^39^ The actuation of three processes at room temperature was fitted using the Specfit or ReactLab software and the observed changes are shown in Figure 3b,c.^49,50^ From that plot, it is clear that the calculated initial spectrum does not match the expected profile for the starting {K^+^} ⊂ (Co^III^_4_/Fe^II^_4_) cube utilized,^40^ indicating that an initial faster process is kinetically lost under the experimental conditions used. Effectively, this initial step can be resolved either by standard mixing at 15 °C, or using stopped-flow techniques at 25 and 35 °C, leading to the identification of four sequential processes corresponding to the stepwise oxidation of the four Fe^II^ centers of the cube. Scheme 2 collects a representation of the sequential redox processes studied, including the known protonation equilibria of the terminal nitrogen atoms of the cyanido groups at pH zero.

**Figure 3:**
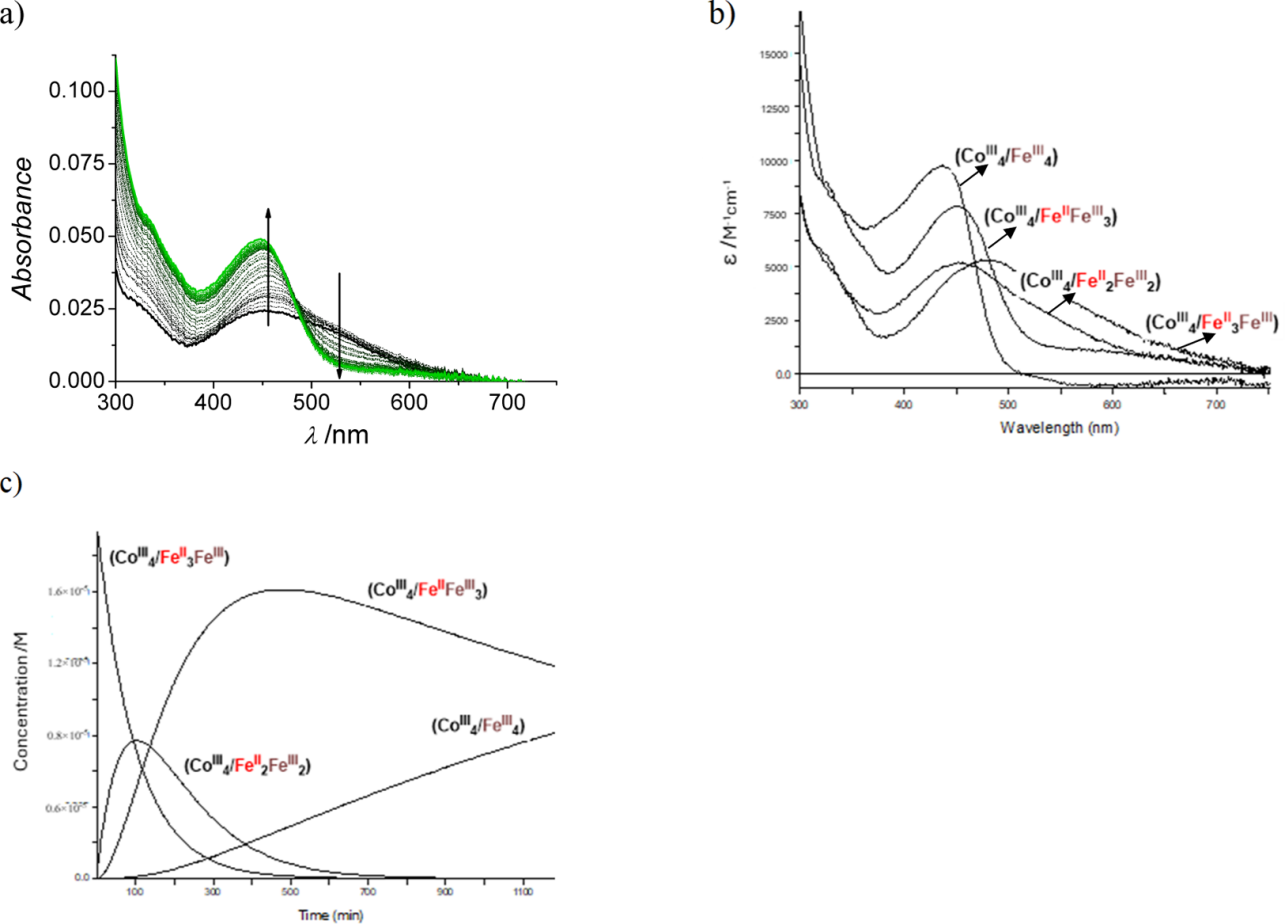
(a) UV–vis time-resolved spectral changes observed for the reaction of an aqueous 1.0 M HCl solution of the potassium-containing (Co^III^_4_/Fe^II^_4_) cubic structure (2 × 10^–5^ M) with a 5 × 10^–3^ M (250-fold) solution of sodium peroxodisulfate at 25 °C (total time 18 h). (b) Specfit-calculated UV–vis spectra of the four species observed on the oxidation reaction indicated in Scheme 2; (c) concentration profiles for the same experiment.

**Scheme 2:**
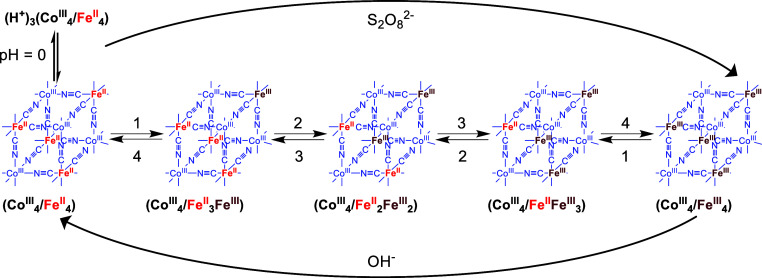


Fortunately, at pH 0 the reaction rates for the four Fe^II^ oxidation steps occur at quite distinct time-scales, allowing time- and temperature-tuning of the reaction rates in order to observe the full multistep reaction and determine the individual rate constants (Figures S2 and S3). All derived rate constants, obtained using the procedures indicated in the Experimental Section, are summarized in Table S1 as a function of both concentration and temperature; it is important to indicate that due to the known/established mathematical ambiguity,^51,52^ there is no unique sequential association between the set of fitted first order rate constants. We have chosen the expected *k*_obs1_ > *k*_obs2_ > *k*_obs3_ > *k*_obs4_ trend, as any other produces much larger intrinsic errors due to the absence of buildup of intermediates. Furthermore, the UV–vis calculated spectra have to be in agreement with the expected and observed trends, which is the case featured in Figures 3 and S5, despite the appearance of formally negative fitted spectra in the zero-absorbance region.

Figure 4a shows representative plots of the observed rate constant (*k*_obs_) for the four oxidation steps at 35 °C at pH 0 for the potassium (Co^III^_4_/Fe^II^_4_) cubic complex, while Figure S4 features the temperature-dependence of the pseudo-first-order rate constants for the first oxidation step as a function of [S_2_O_8_^2–^]. The slopes of all these plots correspond to *k*_et_ × *K*_OS_ at each temperature (see kinetic considerations above), and the Eyring plot of their variation (Figure 4b and eq S2) yields the corresponding thermal activation parameters Δ*H* ^⧧^ and Δ*S* ^⧧^. It is important to indicate that the inherent errors involved in determining *k*_obs#_ for this series of four-step consecutive reactions preclude a fully precise evaluation of the Eyring parameters; nevertheless, the overall trend is clearly defined (see Figure S7). Table 1 summarizes the kinetic data and approximate activation parameters for the processes studied.

**Figure 4:**
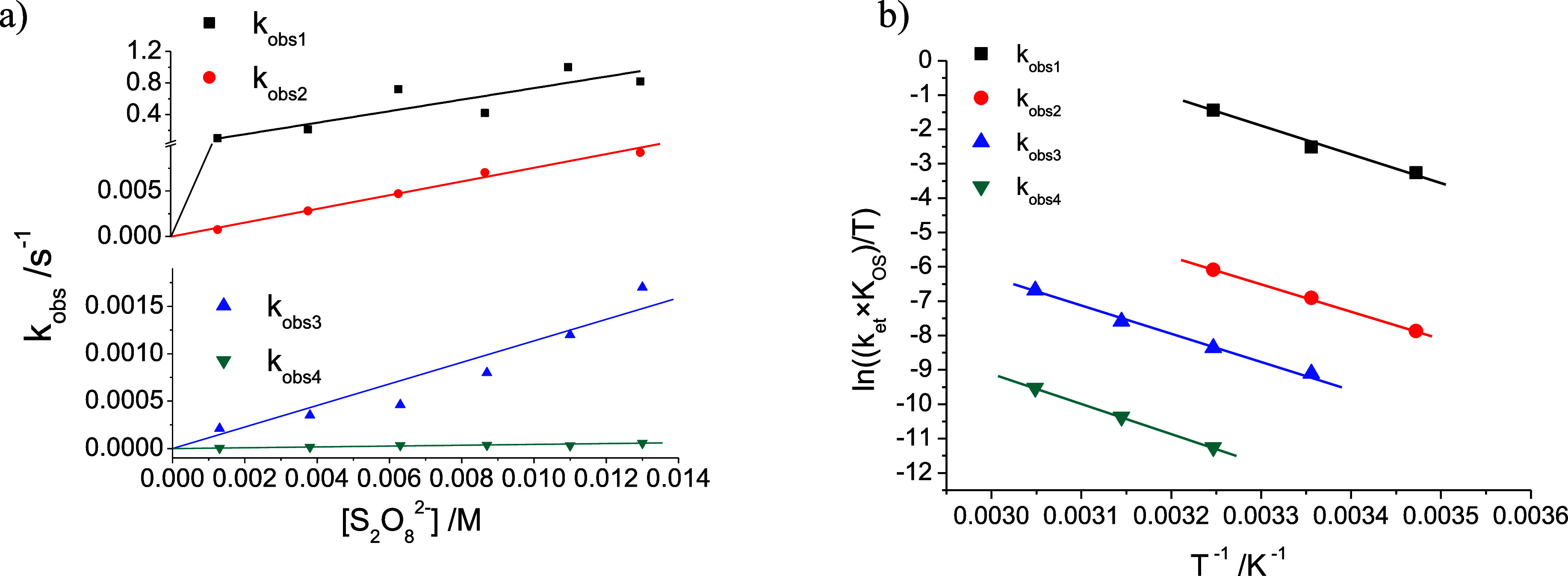
(a) Selected plots of the values of the observed rate constants, derived from the fitting of the time-resolved changes in the UV–vis spectra, for the reaction of {K^+^} ⊂ (Co^III^_4_/Fe^II^_4_) with sodium peroxodisulfate in 1.0 M HCl at 35 °C. (b) Eyring plot for the variation of the slopes of these *k*_obs_ versus [S_2_O_8_^2–^] plots as a function of temperature.

**Table 1:** Kinetic (Extrapolated at 288 K for All Steps) and Thermal Activation Parameters Evaluated for the Reaction of the (Co^III^_4_/Fe^II^_4_) Cubic Structures with Peroxodisulfate

encapsulated unit/medium						
{K^+^}/1.0 M HCl	^288^(*k*_et_ × *K*_OS_)_1_/M^–1^ s^–1^	11 ± 1	(Δ*H* ^⧧^)_1_/kJ mol^–1^	68 ± 8	(Δ*S* ^⧧^)_1_/J K^–1^ mol^–1^	8 ± 28
	^288^(*k*_et_ × *K*_OS_)_2_/M^–1^ s^–1^	0.12 ± 0.1	(Δ*H* ^⧧^)_2_/kJ mol^–1^	66 ± 1	(Δ*S* ^⧧^)_2_/J K^–1^ mol^–1^	–35 ± 3
	^288^(*k*_et_ × *K*_OS_)_3_/M^–1^ s^–1^	0.018 ± 0.001	(Δ*H* ^⧧^)_3_/kJ mol^–1^	62 ± 8	(Δ*S* ^⧧^)_3_/J K^–1^ mol^–1^	–65 ± 27
	^288^(*k*_et_ × *K*_OS_)_4_/M^–1^ s^–1^	0.00054 ± 0.00005	(Δ*H* ^⧧^)_4_/kJ mol^–1^	73 ± 1	(Δ*S* ^⧧^)_4_/J K^–1^ mol^–1^	–56 ± 2
{Na(OH_2_)}^+^/1.0 M HClO_4_	^288^(*k*_et_ × *K*_OS_)_1_/M^–1^ s^–1^	4.4 ± 0.1	(Δ*H* ^⧧^)_1_/kJ mol^–1^	58 ± 8	(Δ*S* ^⧧^)_1_/J K^–1^ mol^–1^	–34 ± 27
	^288^(*k*_et_ × *K*_OS_)_2_/M^–1^ s^–1^	0.74 ± 0.05	(Δ*H* ^⧧^)_2_/kJ mol^–1^	49 ± 2	(Δ*S* ^⧧^)_2_/J K^–1^ mol^–1^	–77 ± 7
	^288^(*k*_et_ × *K*_OS_)_3_/M^–1^ s^–1^	0.077 ± 0.007	(Δ*H* ^⧧^)_3_/kJ mol^–1^	70 ± 11	(Δ*S* ^⧧^)_3_/J K^–1^ mol^–1^	–25 ± 38
	^288^(*k*_et_ × *K*_OS_)_4_/M^–1^ s^–1^	0.0086 ± 0.0006	(Δ*H* ^⧧^)_4_/kJ mol^–1^	60 ± 15	(Δ*S* ^⧧^)_4_/J K^–1^ mol^–1^	–78 ± 51

As seen in Table 1 and Figure 4a, for the fully symmetrical {K^+^} ⊂ (Co^III^_4_/Fe^II^_4_) cube, the second-order rate constant decreases by 1–2 orders of magnitude for each successive oxidation step. This factor is related to increasing entropic requirements, as observed in the parallel fittings in Figure 4b and the data derived shown in Table 1. Given the fact that the values of the second-order rate constant include *k*_et_ and *K*_OS_, both must play a role in determining the observed activation parameters. As the known electrochemistry of the cube shows that each Fe^II^ oxidation step involves an increase of the value of the redox potential by ca. 100–150 mV,^34,36,39,40^ this should be reflected in *k*_et_ according to Marcus theory,^53^ in good agreement with the observed rate constants data. On the other hand, as oxidation of each Fe^II^ decreases by one unit the negative charge of the cubic structure, the value of the outer-sphere pre-equilibrium association with the anionic S_2_O_8_^2–^ oxidant is expected to increase, which is a trend opposite to that observed for the rate constants. Clearly then, the redox potential seems to be the dominant factor governing the experimental trends observed in the values of the rate constants.

The equivalent oxidation reaction of the sodium-encapsulated cube, {Na­(OH_2_)^+^} ⊂ (Co^III^_4_/Fe^II^_4_), was also conducted, given the significant differences observed in its electrochemistry behavior. This difference (also seen for the cube with encapsulated {Li­(OH_2_)^+^} units) can be easily associated with the nonspherical arrangement of the charged {Na­(OH_2_)^+^} unit inside the cage, producing a lack of charge uniformity inside the structure, as shown in Figure 2.^35,36^ Interestingly, for this cube, although four redox steps were also readily observed (see Figures S5 and S6 and Table S1), all of them occurred within a much narrower time span than for the potassium analogue. Eyring plots allow the approximate (see above) evaluation of the corresponding thermal activation parameters collected in Table 1 along with the kinetic data. Similarly to the oxidation of the {K^+^} ⊂ (Co^III^_4_/Fe^II^_4_) cube, the trend in the rate constants of the consecutive steps can be associated mainly to the redox potential, as the values of *K*_OS_ are expected to show the inverse trend (see above). As for the values of the thermal activation parameters, inspection of Figure S7 reveals that, while no clear trend is observed in the activation enthalpy values, the activation entropy becomes increasingly negative as the number of oxidized centers increases, in good agreement with the {K^+^} ⊂ (Co^III^_4_/Fe^II^_4_) cube data. Again, this behavior can be attributed to the decreasing overall negative charge of the cubic structure, a fact that should favor outer-sphere complexation/ordering with the anionic oxidant leading to a higher *K*_OS_ value and a decrease in entropy if solvation remains constant.

In a broader context, the oxidation reactivities of the two cubes (Table 1) imply that the units encapsulated (either {K^+^} or {Na­(OH_2_)^+^}) play a key role in governing the oxidation process. Figure 5 clearly shows that the {Na­(OH_2_)^+^} ⊂ (Co^III^_4_/Fe^II^_4_) compound exhibits a narrower range of rate constants compared with that of the {K^+^} ⊂ (Co^III^_4_/Fe^II^_4_) analogue; furthermore, the third and fourth oxidation step rate constants for the {Na­(OH_2_)^+^} cube are similar to the second and third occurring on the {K^+^} structures. Overall, it is evident that the nature of the encapsulated unit, {K^+^} or {Na­(OH_2_)^+^}, significantly influences the stepwise four-electron oxidation of the Fe^II^ centers, yielding distinct kinetic profiles. Even so, the final nature of the oxidized cubic structures, particularly regarding the retention of the encapsulated {K^+^} or {Na­(OH_2_)^+^} units is not clear, and the study of the oxidized species was also conducted.

**Figure 5:**
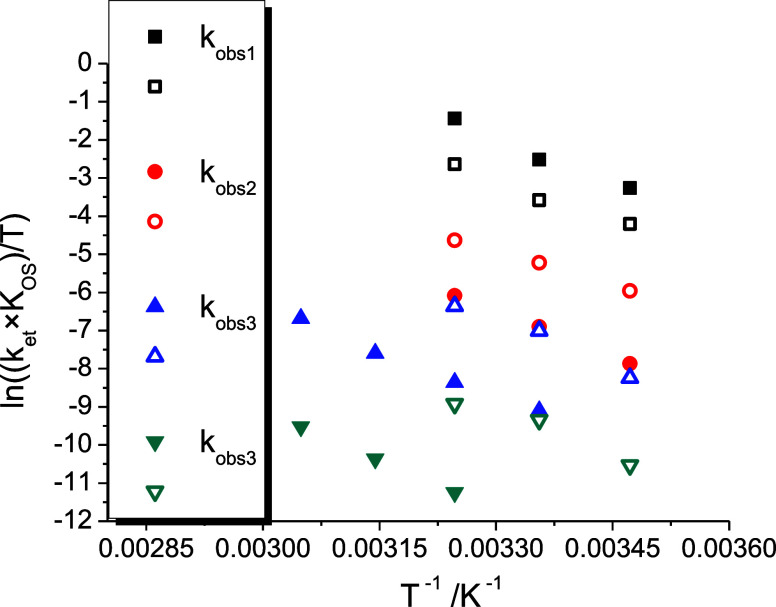
Comparison of the Eyring plots for the variation of the slopes of the *k*_obs_ versus [S_2_O_8_^2–^] plots as a function of temperature for the potassium (full points) and sodium (empty points) (Co^III^_4_/Fe^II^_4_) cubic structures.

The complete oxidation of the {Na­(OH_2_)^+^} ⊂ (Co^III^_4_/Fe^II^_4_) compound was performed as described in the Experimental section. As also noted therein, the solid obtained after its subsequent reduction with NaOH, produced a compound that exhibited a ^1^H NMR spectrum with no signal at 3.87 ppm, indicating the loss of encapsulated water upon full oxidation. The ^23^Na NMR spectrum likewise showed no signal of encapsulated sodium (ca. 5 ppm), while the ^13^C NMR spectrum displayed signals of bridging and terminal cyanido ligands (181.0 and 169.2 ppm, respectively) that match those of the void form of the cubic structure.^35,40^ These data confirm that after the full oxidation of the {Na­(OH_2_)^+^} ⊂ (Co^III^_4_/Fe^II^_4_) compound a release of {Na­(OH_2_)^+^} has occurred, either gradually during the four stepwise oxidation processes or at a specific step. Figure S8 shows the differences in the ^13^C NMR signals of the terminal and bridging cyanido ligands with those reported in the literature, where the {Li­(OH_2_)^+^} and {Na­(OH_2_)^+^} encapsulated species exhibit a larger difference associated with the strain of the cubic structure.^35,40^

### Chemical Reduction

In view of the reversible character of the oxidation process described above, the chemical reduction of the fully oxidized (Co^III^_4_/Fe^III^_4_) cubes at alkaline pHs, a reactivity that has already been described (Equation S3) was pursued.^38,39^ Unfortunately, preparative chemical or electrochemical oxidation to give the sodium or potassium fully oxidized (Co^III^_4_/Fe^III^_4_) cubes, produced mixtures of extremely insoluble species. Moreover, on workup its reversion to the initial reduced (Co^III^_4_/Fe^II^_4_) structures occurs, thereby preventing its isolation and characterization. In fact, the very low concentrations obtained (typically <1 × 10^–5^ M) of oxidized species were insufficient for at least 2-fold dilution-based reverse kinetic studies (see below) taking into account that only a *ca*. 50% (Figure 6b) change in the absorbance values are obtained when compared with the oxidation processes at pH 0 (ca. 250%; Figure 3b). Furthermore, solely the partially oxidized (Co^III^_4_/Fe^III^_3_Fe^II^) structure (see Scheme 2) could be putatively obtained, as described in the Experimental Section, and utilized for this purpose, albeit with some experimental limitations, as discussed below.

**Figure 6:**
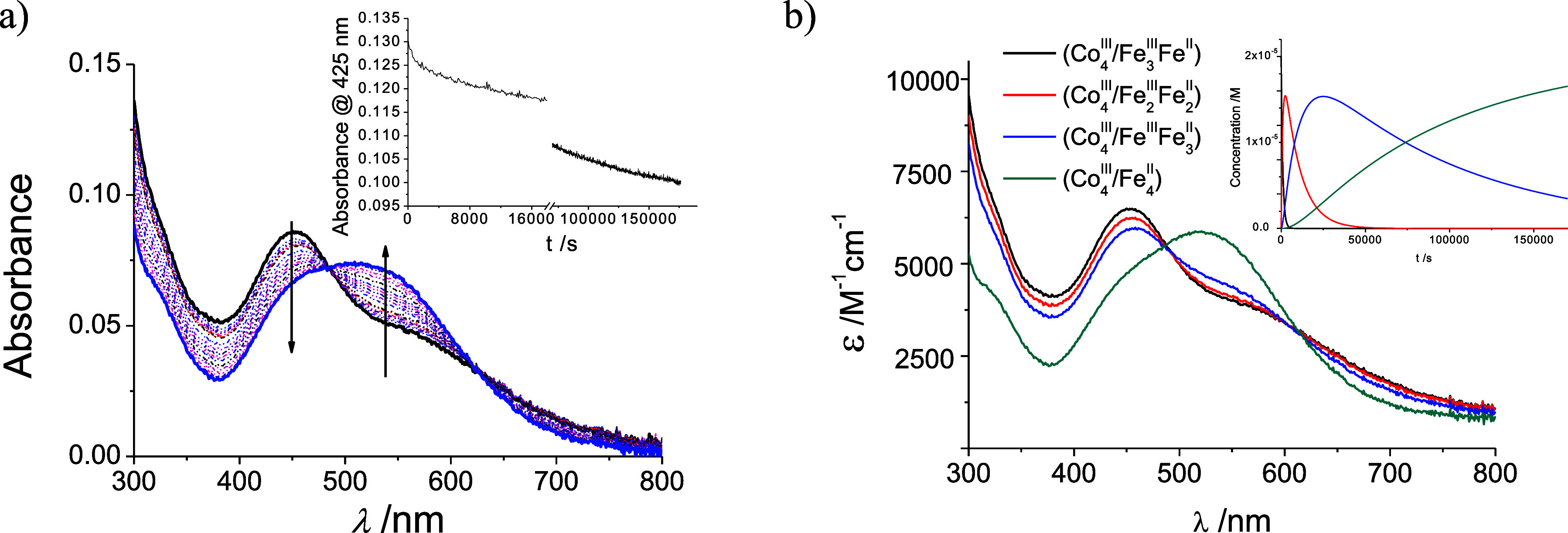
(a) UV–vis time-resolved spectral changes observed on the reduction of a 1 × 10^–5^ M solution of the {Na­(OH_2_)} ⊂ (Co^III^_4_/Fe^III^_3_Fe^II^) species at pH 9.1 at 35 °C (inset corresponds to the absorbance changes at 425 nm; total time 40 h). (b) Specfit-calculated global time-absorbance-wavelength chemometric analysis UV–vis spectra of the four species observed for the reduction processes indicated (inset corresponds to the relative kinetic concentration profiles).

For this putative {Na­(OH_2_)^+^} ⊂ (Co^III^_4_/Fe^III^_3_Fe^II^) or (Co^III^_4_/Fe^III^_3_Fe^II^) structures, three distinct reduction processes are expected when acidic solutions at pH 3 (dilute HCl) are mixed 1:1 with 0.1 M aqueous borax buffer at varying alkalinities (pH = 8.0–9.6). Indeed, the time-resolved UV–vis spectroscopic monitoring of these mixtures (Figure 6) reveals a sequence of three consecutive reaction steps ((Co^III^_4_/Fe^III^_3_Fe^II^) → (Co^III^_4_/Fe^III^_2_Fe^II^_2_) → (Co^III^_4_/Fe^III^Fe^II^_3_) → (Co^III^_4_/Fe^II^_4_) (Scheme 2). As in the case of the oxidation studies described previously, these steps can be quantitatively evaluated using the Specfit or ReactLab software,^49,50^ by appropriate selection of temperature and time-range monitoring.

Figure S9 collects the limited kinetic data obtained for the [OH^–^]-dependence for the three observed processes at different temperatures, while Figure 7 collects the Eyring plots (eq S2) corresponding to the second-order rate constants (M^–1^ s^–1^) derived from the slopes of these plots.^54^ Although thermal activation parameters could not be reliably derived due to the large errors involved, the observed differences in rate constants are clearly attributable to entropic factors, consistent with the trends discussed previously for the oxidation process.

**Figure 7:**
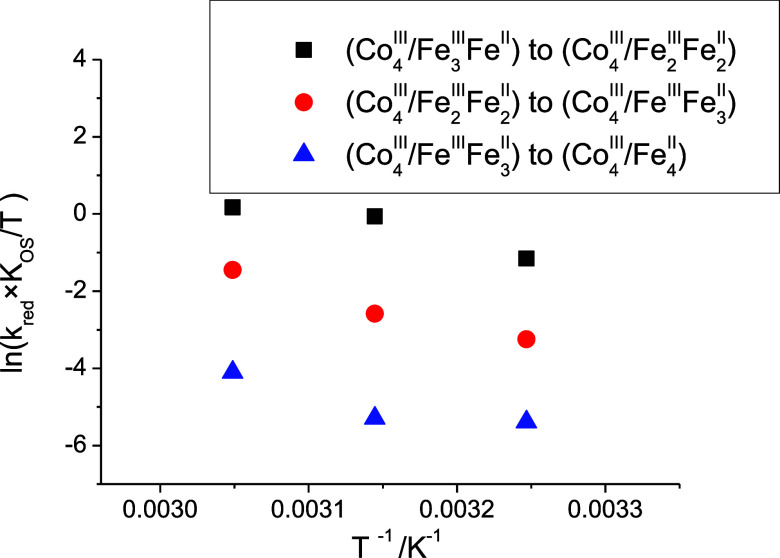
Eyring plots for the variation of the slopes of the *k*_obs_ versus [OH^–^] plots (Figure S9) for the reduction of the {Na­(OH_2_)^+^} ⊂ (Co^III^_4_/Fe^III^_3_Fe^II^) or (Co^III^_4_/Fe^III^_3_Fe^II^) species (Scheme 2) as a function of temperature.

### Electrochemical Behavior of the Redox Process

To assess the stability of the encapsulated cationic units during heterogeneous redox cycling, the electrochemical behavior of the cyanido-bridged {Na­(OH_2_)^+^} ⊂ (Co^III^_4_/Fe^II^_4_) cube was examined by cyclic voltammetry across various supporting electrolyte (NaCl and KCl) concentrations. The objective being to determine whether the encapsulated {Na­(OH_2_)^+^} unit remains within the (Co^III^_4_/Fe^II^_4_) framework through successive oxidation–reduction cycles, particularly given the very distinct electrochemical behavior of the {Na­(OH_2_)^+^} ⊂ (Co^III^_4_/Fe^II^_4_) and {K^+^} ⊂ (Co^III^_4_/Fe^II^_4_) cubes.

Figure 8 displays the cyclic voltammograms of the {Na­(OH_2_)^+^} ⊂ (Co^III^_4_/Fe^II^_4_) species recorded in the absence of supporting electrolyte (a), in 1.0 M NaCl (b), and in saturated NaCl solution (c). Comparison with the {K^+^},{Rb^+^}, and {Cs^+^} encapsulated analogues (Figure S10) reveals very distinct features; in a saturated NaCl solution (Figure 8c), the {Na­(OH_2_)^+^} ⊂ (Co^III^_4_/Fe^II^_4_) species exhibits an electrochemical behavior with a large peak-to-peak separation, which is maintained when undergoing multiple redox cycles. However, in the absence of a supporting electrolyte, the voltammogram progressively evolves upon repetitive cycling toward the electrochemically reversible profile characteristic of the void, {K^+^}, {Rb^+^}, and {Cs^+^}­encapsulated species. In KCl medium (1.0 M KCl, Figure S11), upon the second oxidation cycle, the voltammogram’s profile matches that of the {K^+^} encapsulated species.

**Figure 8:**
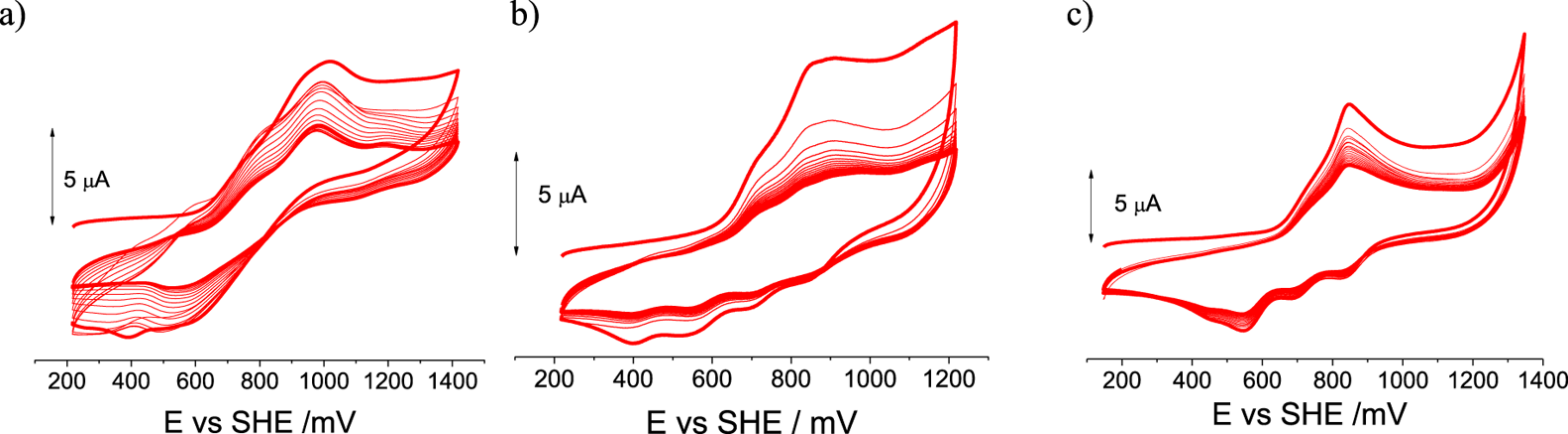
Cyclic voltammograms of a 1 mM aqueous solution of {Na­(OH_2_)^+^} ⊂ (Co^III^_4_/Fe^II^_4_) with (a) no supporting electrolyte; (b) in 1.0 M NaCl solution; (c) in saturated NaCl solution. Recorded at a scan rate of 100 mV s^–1^, at a glassy carbon working electrode, and at room temperature.

This behavior indicates that, under conditions of low sodium concentration, the {Na­(OH_2_)^+^} unit is expelled from the cubic framework during redox cycling, resulting in a void symmetrical cube with an electrochemically reversible voltammetric profile (Figure S10).^40^ This parallels observations from chemical oxidation procedures where the value of [Na^+^] is kept in the 0.1–0.5 M range. Conversely, the retention of the initial CV profile under saturated NaCl conditions (Figure 8c) implies that the expulsion process is reversible in the presence of an excess of sodium, a fact that agrees with the significant outer-sphere complexation previously observed between Na^+^ and the {Na­(H_2_O)^+^} ⊂ (Co^III^_4_/Fe^II^_4_) cube on the sodium-to-potassium exchange process already studied (*K*_OS_ ca. 10 M^–1^).^40^ Moreover, the results in 1.0 KCl media (Figure S11) indicate that the exchange of {Na­(OH_2_)^+^} by {K+} is occurring as expected.

Since the potential expulsion of the {K^+^} unit cannot be monitored spectrophotometrically (^39^K NMR produces very wide signals),^55^ and no entry of external sodium is observed under the chemical redox conditions used (see before), an electrochemical experiment were specifically designed. While standard CVs of the {K^+^} ⊂ (Co^III^_4_/Fe^II^_4_) cube in 0.1 M NaCl or KCl show characteristically reversible CV profiles (Figure S10), the experiments carried out in 1.0 M NaCl (Figure S12) do not produce any significant changes. This indicates that there is no exchange or void cube formation when redox cycling is conducted on the {K^+^} encapsulated species. This is in good agreement with the fact that the larger spherical cations {K^+^}, {Rb^+^} and {Cs^+^} remain encapsulated during cycling, consistently with their greater size and the stability of the self-assembled cubic structures previously reported.^35^

## Conclusions

This study provides the first full kinetic picture of the four-electron redox cycling within discrete Prussian-Blue-Analogue molecular cubic structures, {M^+^} ⊂ (Co^III^_4_/Fe^II^_4_)}. The Fe^II^ → Fe^III^ chemical oxidation by peroxodisulfate proceeds through four kinetically distinct steps with very different rate constants that increase with increasing redox potential of the process and with decreasing the charge of the species. These trends show that electron transfer energetics outweigh outer-sphere ion pairing for these redox processes. Similarly, chemical reduction of the oxidized species confirms that the multistep process is reversible and shaped by significant entropic effects.

The encapsulated cationic units play a crucial role in the redox behavior. While the {K^+^} encapsulated cube displays widely separated rate constants keeping its guest throughout oxidation, the more strained {Na­(OH_2_)^+^} analogue oxidizes within a narrow kinetic range. Moreover, full oxidation results in expulsion of the inner {Na­(OH_2_)^+^} unit, to give a void structure, as verified by NMR analysis. Electrochemical cycling mirrors this trend: under low [Na^+^] as electrolyte, the {Na­(OH_2_)^+^} framework’s voltammetric profile becomes that of the void cube, while high [K^+^] also alters the redox profile producing that of the {K^+^} encapsulated species; the original profile is retained in saturated NaCl conditions. In contrast, the {K^+^} encapsulated cube’s voltammetric profile remains unchanged even under saturated [Na^+^] conditions. Overall, these results reveal a direct link between electron transfer and encapsulated cation-PBA cage behavior and sets a foundation for designing stimuli-responsive ion-storage materials and redox mediators.

## Experimental Section

### Preparation of the Complexes

The {Na­(OH_2_)^+^} ⊂ [{Co^III^(Me_3_-tacn)}_4_{Fe^II^(CN)_6_}_4_]} and {K^+^} ⊂ [{Co^III^(Me_3_-tacn)}_4_{Fe^II^(CN)_6_}_4_]} complexes were prepared according to previously established procedures.^36,40^ As derived from the exchange reactivity described, all manipulations of the {Na­(OH_2_}^+^} ⊂ [{Co^III^(Me_3_-tacn)}_4_{Fe^II^(CN)_6_}_4_]} species have been conducted in strict absence of potassium cations. In contrast, for the {K^+^} ⊂ [{Co^III^(Me_3_-tacn)}_4_{Fe^II^(CN)_6_}_4_]} species, manipulation could proceed without restrictive presence of sodium cationic compounds. ^1^H and ^13^C NMR spectra were recorded on a Bruker-400Q or on a Bruker-500 spectrometers at 25 °C and ^23^Na NMR spectra were recorded on a Bruker-500 instrument at the Unitat de RMN d'Alt Camp de la Universitat de Barcelona.

The partially oxidized {Na­(OH_2_)^+^} ⊂ (Co^III^_4_/Fe^II^_4_) cubic molecular units were prepared by addition to a saturated solution of the original cubes in 1 × 10^–3^ M HCl solid sodium peroxodisulfate to reach a 0.05 M concentration. Upon addition, an off-yellow precipitate formed immediately; the suspension was stirred for 5–6 h, then centrifuged and repeatedly washed with 1 × 10^–3^ M HCl to ensure no excess sodium peroxodisulfate remained. The UV–vis spectrum of the final complex in 1 × 10^–3^ M HCl solution still shows the signature of some remaining MMCT band; this suggests that the precipitate should correspond to the partially oxidized {Na­(OH_2_)^+^} ⊂ (Co^III^_4_/Fe^III^_3_Fe^II^) or (Co^III^_4_/Fe^III^_3_Fe^II^) structure.

The complete oxidation of {Na­(OH_2_)^+^} ⊂ (Co^III^_4_/Fe^II^_4_) compound was performed *in situ* by stirring a solution of the complex in a 0.1 M aqueous Na_2_S_2_O_8_ medium for 36 h at 40 °C. After this time, the reaction mixture was evaporated to dryness, to yield a pale-yellow residue, which was chromatographed repetitively (3–4 times) on a Sephadex G-25 size exclusion column to remove excess oxidant. The final solution was evaporated to dryness, and the resulting light brown solid was suspended in 0.05 M aqueous NaOH overnight to obtain the initial reduced species, as previously described.^38,39^ Subsequent anionic Sephadex DEAE chromatography afforded a main purple band, which was eluted with aqueous 0.2 M NaClO_4_, evaporated to dryness and desalted by repeated (3–4 times) Sephadex G-25 size exclusion chromatography. The final solid, obtained after evaporation of the eluate, exhibited a ^1^H NMR spectrum with no signal at 3.87 ppm, indicating the loss of encapsulated water upon full oxidation.

### Kinetics

The kinetic profiles for the reactions were followed by UV–vis spectroscopy in the 700–300 nm range on HP8453A or Cary50 instruments equipped with thermostated multicell transports. Runs with *t*_1/2_ < 10 s were performed using an Applied Photophysics SX20 MV Stopped-flow instrument with photodiode array detection (J&M TIDAS). The concentration of the solutions of the (Co^III^_4_/Fe^II^_4_) cubes was kept within the 1–3 × 10^–5^ M range, while the oxidant varied from 0.0005 to 0.015 M and that of the reductant (OH^–^) from 1 × 10^–6^ to 30 × 10^–6^ M in borax buffer at 1.0 M ionic strength. To prevent precipitation, oxidation reactions were conducted in 1.0 M HCl for the potassium-containing structures and in 1.0 M HClO_4_ for those containing sodium. Reduction experiments were always performed using 0.001 M HCl stock solutions, filtered through Celite prior to use, by adding the proper amount of borax buffer. The general kinetic methodology has been described previously.^37–39,56^

The values of the observed rate constants were derived from the time-resolved absorbance changes using Specfit or ReactLab.^57^ The process involves minimization of errors in a chemometric Global Analysis on the time-absorbance-wavelength data and further fitting to an estimated reaction sequence. The process produces the best fitting to the data and the calculated spectra of the intervening species and time-resolved relative concentration profiles.^58,59^ When the raw fitting gave rate constant values that do not agree with the expected *k*_obs1_ > *k*_obs2_ > *k*_obs3_... trend, they were reordered to fit the indicated sequence.^60^ As the full processes studied involve four (oxidation) or three (reduction) consecutive steps, the errors of the methods were considered too large and the monitoring time interval, temperature and oxidant concentrations were tuned to observe solely two or three steps at a time. Figures S2 and S3 collect a selected sample of such fitting procedures. The agreement with the data from different sets of experiments was considered as proof of its validity. In general, the data indicated in Table S1 correspond to the average of 3–4 measures and/or measuring conditions with an average error of 20%. All postrun fittings were carried out using the standard available commercial programs and the estimated errors fall within the 10–15% range.

### Electrochemistry

Cyclic voltammetry measurements were performed at 23 °C using either a SP-150 (BioLogic, France) or a PGSTAT204 potentiostat. A conventional three-electrode configuration was employed, consisting of a glassy carbon working electrode (3 mm diameter), a platinum wire counter electrode, and a Ag/AgCl reference electrode (saturated KCl or NaCl, depending on the cube analyzed). Before each measurement, the working electrode was polished on a microcloth polishing pad with a 0.05 μm alumina slurry, then thoroughly rinsed with deionized water and ethanol, and sonicated briefly to remove residual alumina. Before use, the polished electrode was dried under a gentle nitrogen stream. Aqueous sample solutions of the cubes (typically 1 × 10^–3^ M) were prepared using NaCl or KCl (1.0 M–saturated) as supporting electrolyte. Solutions were purged with high-purity N_2_ for at least 10 min before data collection, a nitrogen atmosphere was maintained over the solution during measurements.

## Supplementary Material



## Notes

Time-speciation fittings of the reactions studied, plots of the observed rate constants versus the concentration of oxidant or reductant. Trends in the thermal activation parameters and in the ^13^C NMR chemical shift for the different structures. First-order rate constants for the reactions studied as a function oxidant or reductant concentration and temperature. Cyclic voltammogram of the {Na­(OH_2_)^+^} and {K^+^}-cubes under different NaCl or KCl electrolyte concentrations (PDF)
