# Supplementary material for: Kinetico-Mechanistic Study of the Chemical Redox Cycling of Cubic PBA {M+} ⊂ (CoIII 4/FeII 4) Structures
Source: Inorg Chem. 2026 May 8;65(20):10925–35. doi: 10.1021/acs.inorgchem.5c05805 (PMC13213909; doi:10.1021/acs.inorgchem.5c05805)
Supplement: Supplementary file 1 [file ic5c05805_si_001.pdf]

SUPPORTING INFORMATION

Kinetico-mechanistic study of the chemical redox cycling of cubic PBA $\{M^+\}\subset(Co^{III}_4/Fe^{II}_4)$ structures

Miguel A. Gonzálvez,[a,b*] *Montserrat Ferrer,*[b,c] *and Manuel Martínez*[b,c*]

[a] CNRS/Université de Toulouse, Laboratoire Hétérochimie Fondamentale et Appliquée (LHFA, UMR 5069), 118 Route de Narbonne, 31062 Toulouse Cedex 09, France

[b] Secció de Química Inorgànica, Departament de Química Inorgànica i Orgànica. Universitat de Barcelona, Martí i Franquès 1-11, 08028 Barcelona, Spain

[c] Institute of Nanoscience and Nanotechnology (IN2UB), Universitat de Barcelona, 08028 Barcelona, Spain

miguel.gonzalvez-noguera@utoulouse.fr, manel.martinez@qi.ub.edu

Time-speciation fittings of the reactions studied, plots of the observed rate constants versus the concentration of oxidant or reductant. Trends in the thermal activation parameters and in the ^{13}C NMR chemical shift for the different structures. First-order rate constants for the reactions studied as a function oxidant or reductant concentration and temperature. Cyclic voltammogram of the $\{Na(OH_2)^+\}$ and $\{K^+\}$-cubes under different NaCl or KCl electrolyte concentrations. Relevant equations utilised for the kinetic and activation parameters.

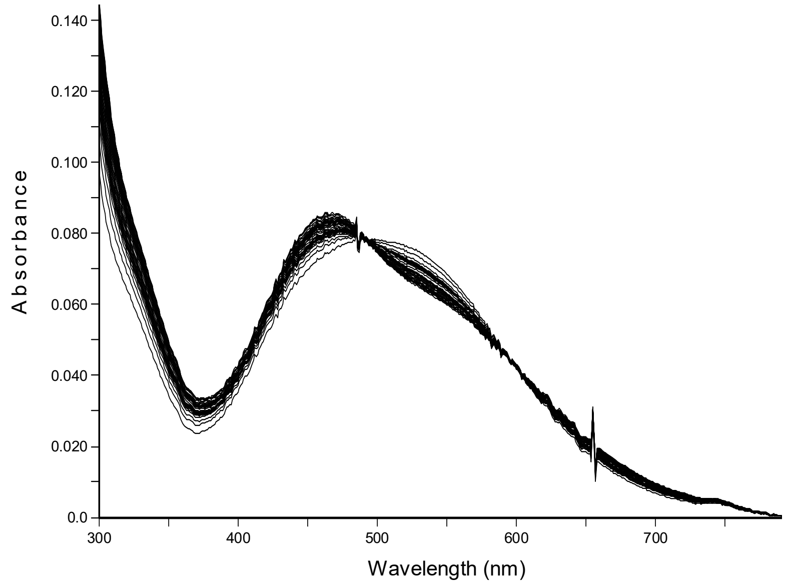

Figure S1.- UV-Vis time-resolved spectral changes observed on the reaction of a borax-buffered solution of the $\{K^+\}\subset(Co^{III}_4/Fe^{II}_4)$ cubic structure (2×10^{-5} M) with a 0.10 M (5000 fold) solution of sodium peroxodisulfate at 25 ºC.

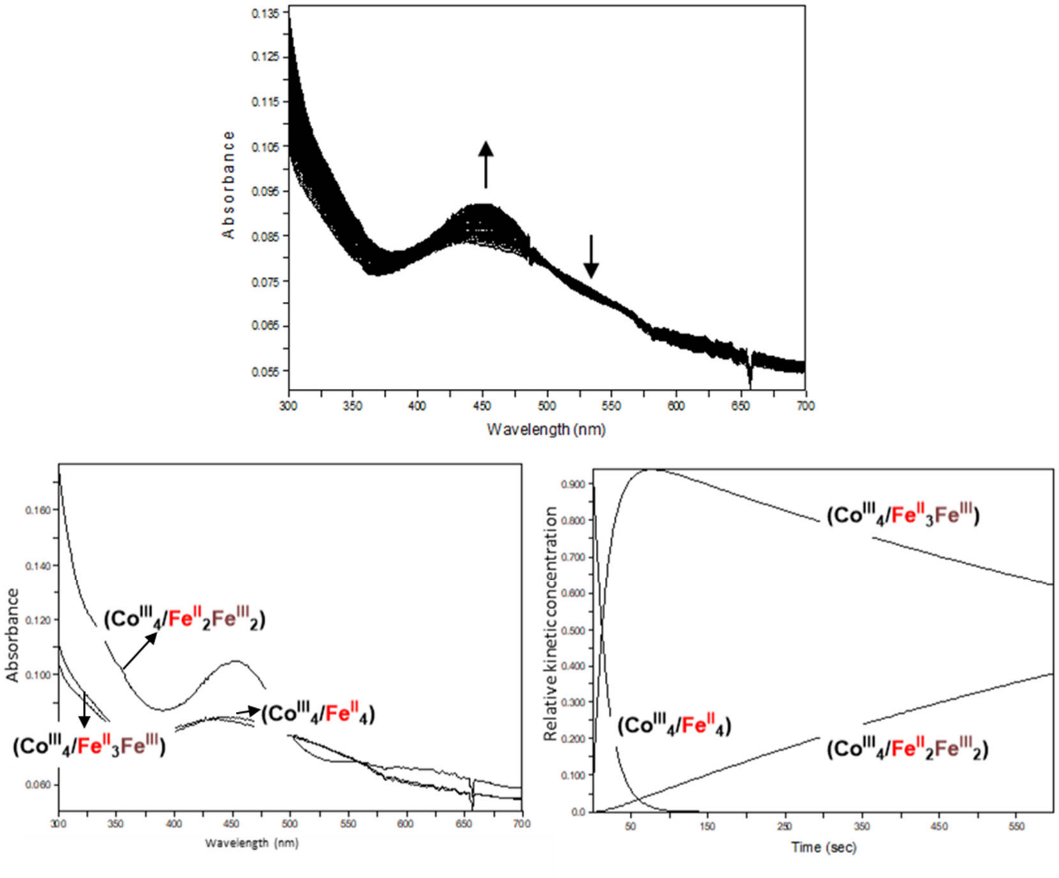

Figure S2.- UV-Vis spectral time-resolved changes observed on the reaction of an aqueous 1.0 M HCl solution of the $\{K^+\}\subset(Co^{III}_4/Fe^{II}_4)$ cubic structure (2×10^{-5} M) with a 0.011 M solution of sodium peroxodisulfate at 45 ^{o}C (top) and results of the time-resolved spectra (bottom left) and concentration (bottom right) fitting to a A→B→C (k_{obs1} and k_{obs2}) reaction sequence.

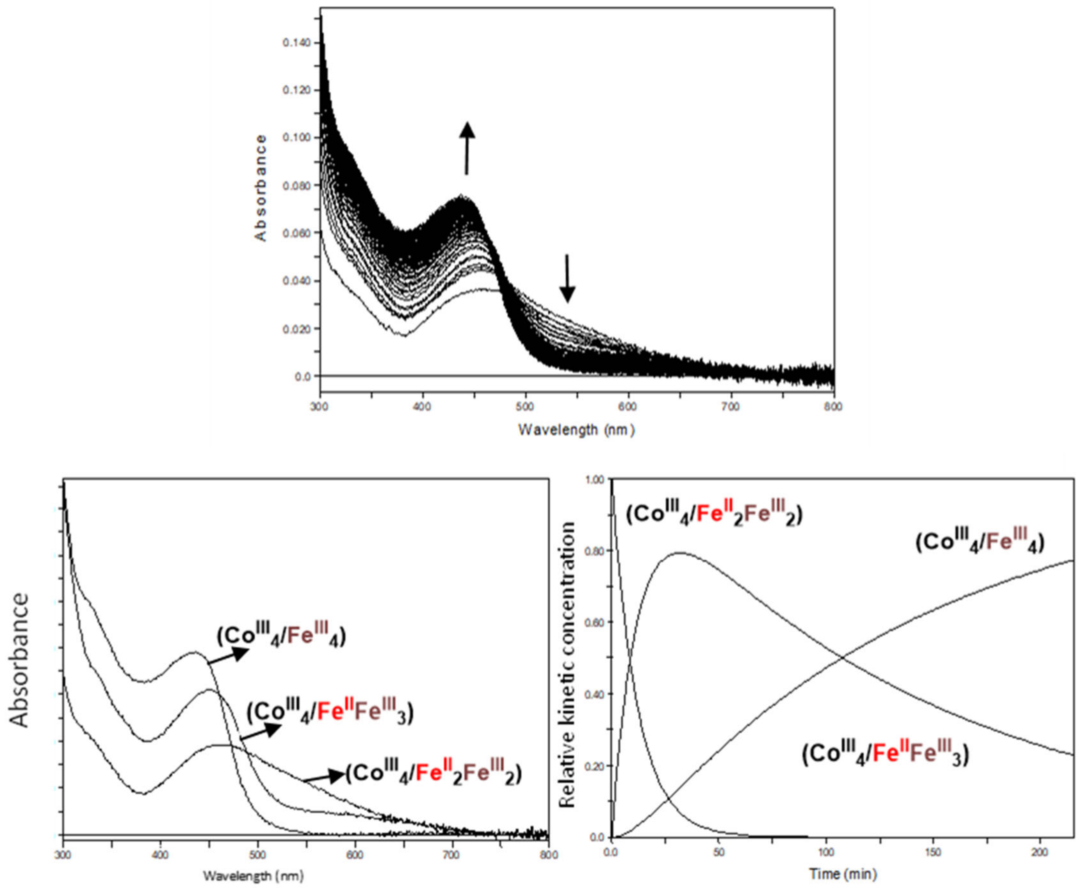

Figure S3.- UV-Vis time-resolved spectral changes observed on the reaction of an aqueous 1.0 M HCl solution of the $\{K^+\}\subset(Co^{III}_4/Fe^{II}_4)$ cubic structure (2×10^{-5} M) with a 0.0063 M solution of sodium peroxodisulfate at 15 ºC (top) and results of the time-resolved spectra (bottom left) and concentration (bottom right) fitting to a A→B→C (k_{obs3} and k_{obs4}) reaction sequence.

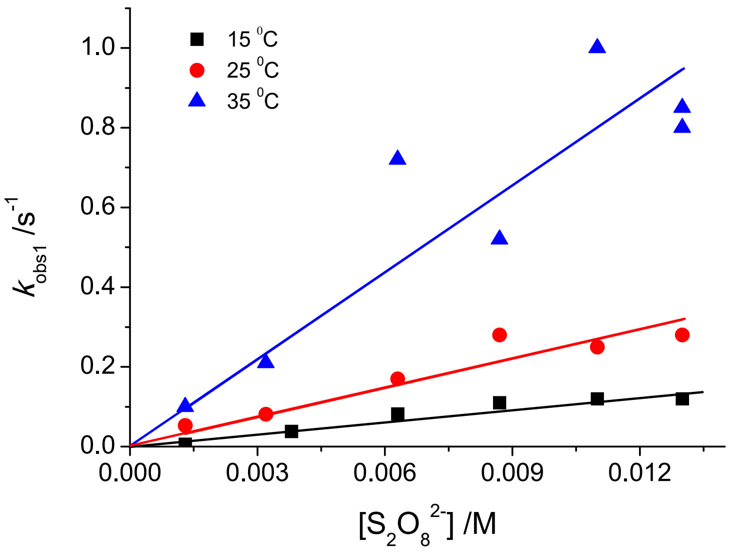

Figure S4.- Plots of the values of the observed rate constants for the first step (Scheme 2) of the oxidation reaction of the potassium (Co^{III}_4/Fe^{II}_4) cubic structure with sodium peroxodisulfate in 1.0 M HCl at different temperatures.

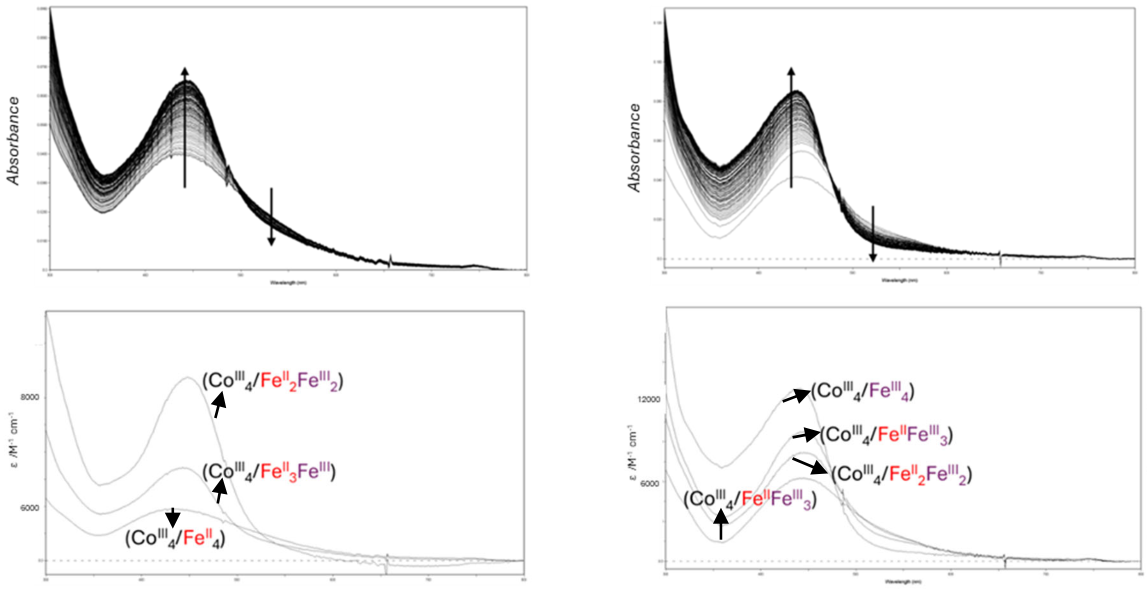

Figure S5.- UV-Vis spectral time-resolved changes observed on the reaction of an aqueous 1.0 M $HClO_4$ solution of the $\{Na(OH_2)^+\}\subset(Co^{III}_4/Fe^{II}_4)$ cubic structure (2×10^{-5} M) with a 0.0013 (left) and 0.0038 M (right) solution of sodium peroxodisulfate at 15 ºC and results of the time-resolved spectra fitting to the respective reactions sequence.

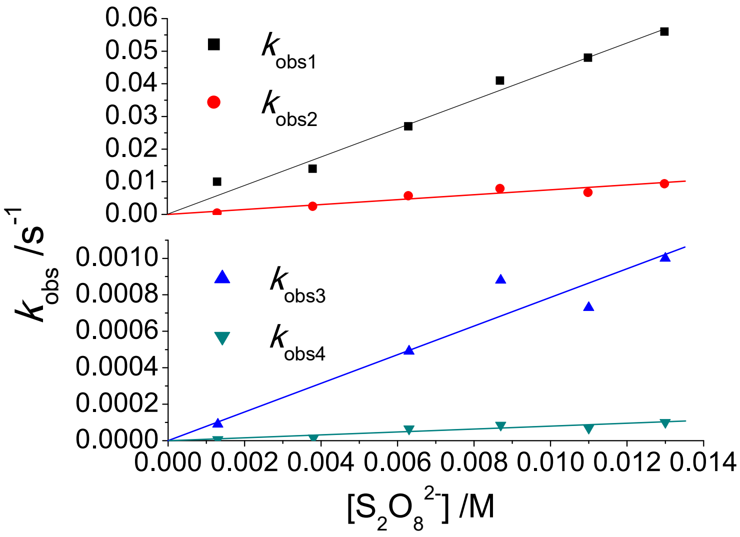

Figure S6.- Plots of the values of the observed rate constants, derived from the fitting of the time-resolved changes in the UV-Vis spectra, for the reaction of the sodium-containing (Co^{III}_4/Fe^{II}_4) cubic structure with sodium peroxodisulfate in 1.0 M $HClO_4$ at 15 ºC (Scheme 2).

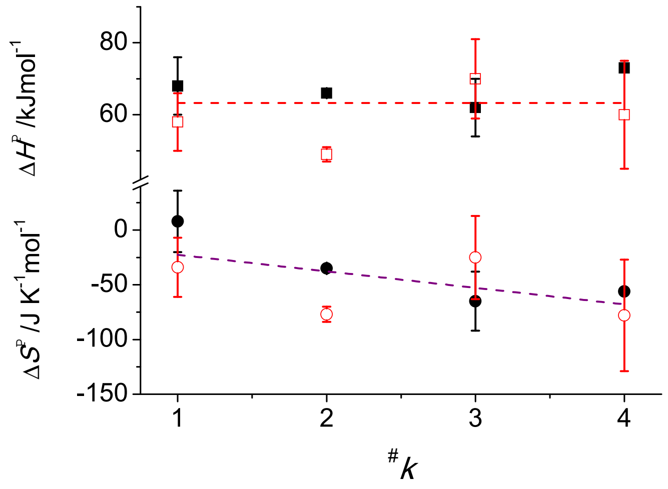

Figure S7.- Plot of the enthalpy and entropy data from Table 1 *versus* the sequential order (Scheme 2) of the oxidation rate constant determined; full points correspond to $\{K^+\}\subset(Co^{III}_4/Fe^{II}_4)$ and empty points to $\{Na(H_2O)^+\}\subset(Co^{III}_4/Fe^{II}_4)$.

a) b)

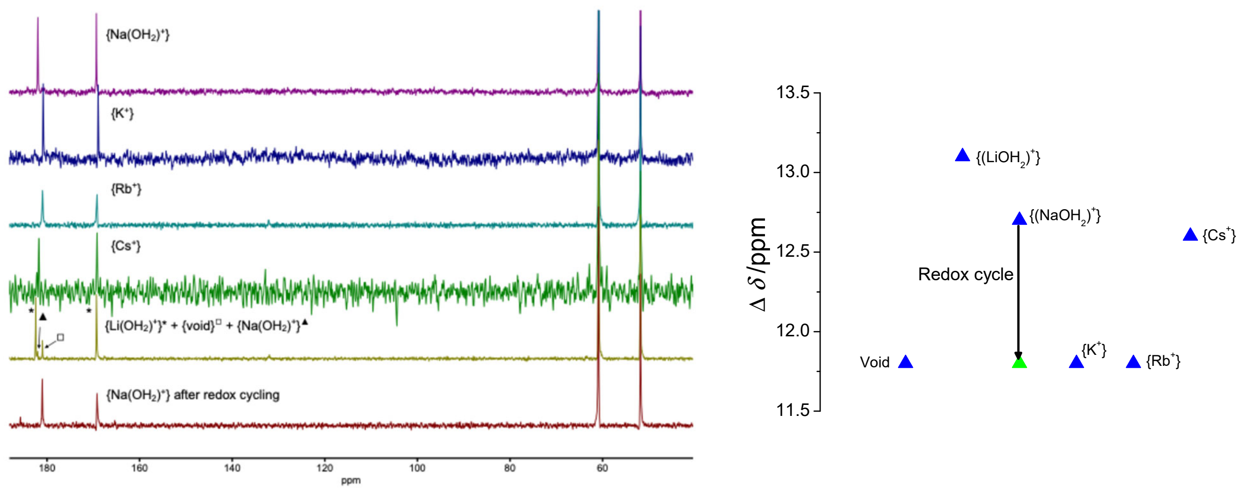

Figure S8.- a) ^{13}C NMR spectra off all the different cubic structures studied; b) Plot of the differences of the signal between the bridging and the terminal cyanido groups in the $\{M^+\}\subset(Co^{III}_4/Fe^{II}_4)$ complexes in this work.

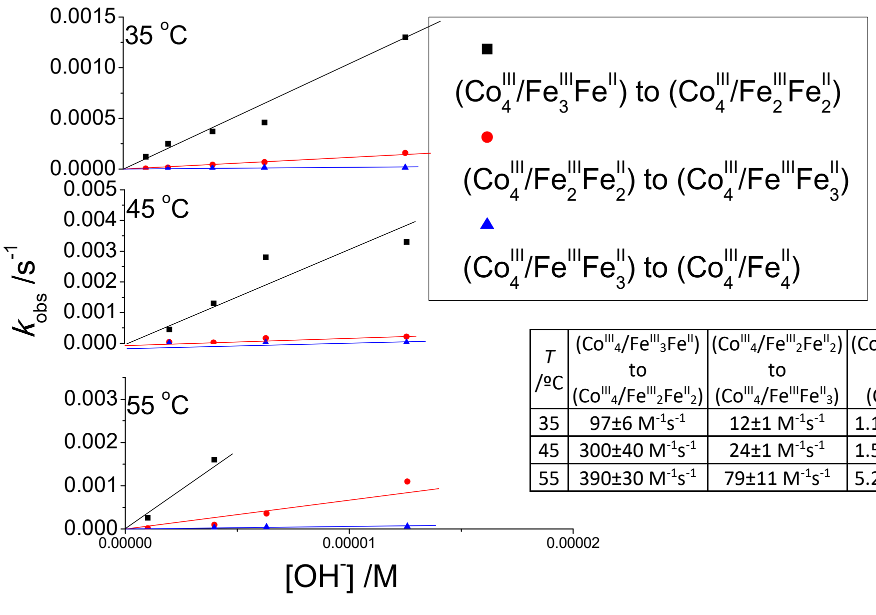

T /ºC	$(Co^{III}_4/Fe^{III}_3Fe^{II})$ to $(Co^{III}_4/Fe^{III}_2Fe^{II}_2)$	$(Co^{III}_4/Fe^{III}_2Fe^{II}_2)$ to $(Co^{III}_4/Fe^{III}Fe^{II}_3)$	$(Co^{III}_4/Fe^{III}Fe^{II}_3)$ to (Co^{III}_4/Fe^{II}_4)
35	97±6 $M^{-1}s^{-1}$	12±1 $M^{-1}s^{-1}$	1.1±0.2 $M^{-1}s^{-1}$
45	300±40 $M^{-1}s^{-1}$	24±1 $M^{-1}s^{-1}$	1.5±0.3 $M^{-1}s^{-1}$
55	390±30 $M^{-1}s^{-1}$	79±11 $M^{-1}s^{-1}$	5.2±0.8 $M^{-1}s^{-1}$

Figure S9.- Plots of the values of the observed rate constants, derived from the fitting of the time resolved changes in the UV-Vis spectra, for the reduction reaction of the $\{Na(OH_2)^+\}\subset(Co^{III}_4/Fe^{III}_3Fe^{II})$ cubic structure (Scheme 2) by OH^- at different temperatures.

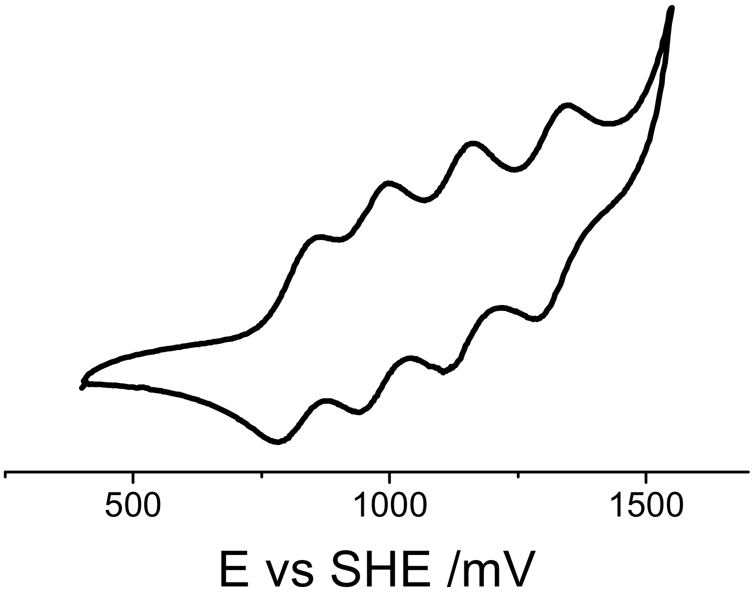

Figure S10.- Typical cyclic voltammogram of the void, $\{K^+\}$, $\{Rb^+\}$, and $\{Cs^+\}$-encapsulated cubic structures, from references [1-3].

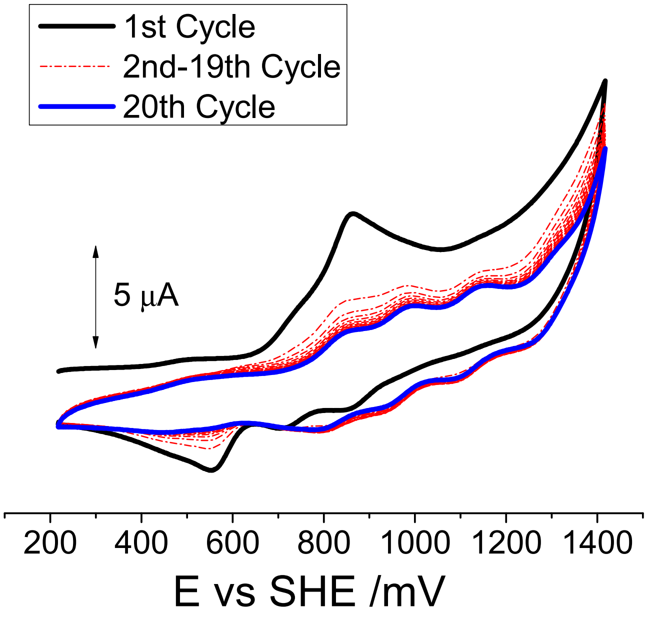

Figure S11.- Cyclic voltammogram (20 cycles) of a 1 mM solution of the $\{Na(OH_2)^+\}\subset(Co^{III}_4/Fe^{II}_4)$ cubic structure in 1.0 M KCl, recorded at a glassy carbon electrode, scan rate 100 mV s^{-1}, room temperature.

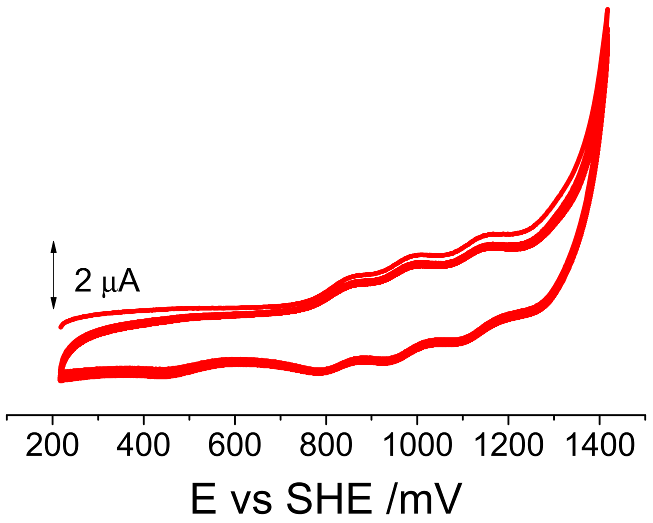

Figure S12.- Cyclic voltammogram (20 cycles) of a 1 mM solution of the $\{K^+\}\subset(Co^{III}_4/Fe^{II}_4)$ cubic structure in 1.0 M NaCl, recorded at a glassy carbon electrode, scan rate 100 mV s^{-1}, room temperature.

Equation S1.- Redox Eigen-Wilkins[4] kinetic mechanism proposed for each single step of the type of oxidation reactions studied under pseudo-first order conditions of excess oxidant (from references [5, 6]). The $\{Co^{III}/Fe^{II}\}^-$ units refer to any of those contained in the cubic species (4 in total) still bearing a Fe^{II} cation.

$$\{Co^{III}/Fe^{II}\}^- + S_2O_8^{2-} \underset{}{\overset{K_{OS}}{\rightleftharpoons}} \{\{Co^{III}/Fe^{II}\}^-;S_2O_8^{2-}\}$$

$$\{\{Co^{III}/Fe^{II}\}^-;S_2O_8^{2-}\} \xrightarrow{k_{ox}} \{Co^{III}/Fe^{III}\} + SO_4^{2-} + SO_4^{\cdot -}$$

$$\{Co^{III}/Fe^{II}\}^- + SO_4^{\cdot -} \xrightarrow{fast} \{Co^{III}/Fe^{III}\} + SO_4^{2-}$$

$$k_{obs} = \frac{k_{ox}\,K_{OS}\,[S_2O_8^{2-}]}{1 + K_{OS}[S_2O_8^{2-}]}$$

→ k_{ox} at large $[S_2O_8^{2-}]$ when K_{OS} is large

→ $k_{ox}\,K_{OS}\,[S_2O_8^{2-}]$ if K_{OS} is small

Equation S2.- Eyring equation according to the model of the transition state in an activated complex.[4, 7]

$$v = k\,[\{AB\}^{\ddagger}]$$

$$A + B \overset{K^{\ddagger}}{\rightleftharpoons} \{AB\}^{\ddagger} \xrightarrow{k} \text{Products}$$

$$K^{\ddagger} = \frac{[\{AB\}^{\ddagger}]}{[A]\,[B]} \qquad k = \frac{k_b\,T\,K^{\ddagger}}{h}$$

$$\Delta G^{\ddagger} = -RT\ln K^{\ddagger} = \Delta H^{\ddagger} - T\Delta S^{\ddagger}$$

$$\ln\left(\frac{k}{T}\right) = \ln\left(\frac{k_b}{h}\right) + \frac{\Delta S^{\ddagger}}{R} - \frac{\Delta H^{\ddagger}}{R}\,\frac{1}{T}$$

Eyring linearised plot

Equation S3 .- Redox Eigen-Wilkins[4] kinetic mechanism proposed for each single step of the reduction type of reactions studied under pseudo-first order conditions of excess reductant (references [5, 6]).

$$\{Co^{III}/Fe^{III}\} + OH^- \underset{}{\overset{K_{OS}}{\rightleftharpoons}} \{\{Co^{III}/Fe^{III}\}^-;OH^-\}$$

$$\{\{Co^{III}/Fe^{III}\}^-;OH^-\} \xrightarrow{k_{red}} \{Co^{III}/Fe^{II}\}^- + OH^{\cdot}$$

$$2\ OH^{\cdot} \xrightarrow{fast} H_2O_2$$

$$k_{obs} = \frac{k_{red}\, K_{OS}\, [OH^-]}{1 + K_{OS}[OH^-]} \longrightarrow k_{obs} = k_{ox}\, K_{OS}\, [OH^-] \quad \text{when } K_{OS} \text{ is small}$$

Table S1.- Values of the observed rates constants derived as indicated in the experimental section, for the (Co^{III}_4/Fe^{II}_4) cubic structures and conditions studied. Slow or fast indicates that the process cannot be reliably monitored under the conditions used in the measure, of either concentration or temperature. Typical errors are with in the 20 % range.

OXIDATION							
Encapsulated unit	Medium	T /ºC	$[S_2O_8^{2-}]$ /M	k_{obs1} /s^{-1}	k_{obs2} /s^{-1}	k_{obs3}”” /s^{-1}	k_{obs4} /s^{-1}
$\{K^+\}$	1.0 M HCl	15	1.3×10^{-2}	1.2×10^{-1}	1.2×10^{-3}	[a]slow	[a]slow
			1.1×10^{-2}	1.2×10^{-1}	1.3×10^{-3}	[a]slow	[a]slow
			8.7×10^{-3}	1.1×10^{-1}	1.2×10^{-3}	[a]slow	[a]slow
			6.3×10^{-3}	8.2×10^{-2}	6.0×10^{-4}	[a]slow	[a]slow
			3.8×10^{-3}	3.8×10^{-2}	4.8×10^{-4}	[a]slow	[a]slow
			1.3×10^{-3}	5.8×10^{-3}	[a]slow	[a]slow	[a]slow
		25	1.3×10^{-2}	2.8×10^{-1}	4.0×10^{-3}	5.0×10^{-4}	[a]slow
			1.1×10^{-2}	2.5×10^{-1}	3.2×10^{-3}	3.5×10^{-4}	[a]slow
			8.7×10^{-3}	2.8×10^{-1}	2.0×10^{-3}	2.5×10^{-4}	[a]slow
			6.3×10^{-3}	1.7×10^{-1}	1.5×10^{-3}	1.5×10^{-4}	[a]slow
			3.2×10^{-3}	8.1×10^{-2}	--	--	--
			3.8×10^{-3}	--	1.0×10^{-3}	1.0×10^{-4}	[a]slow
			1.3×10^{-3}	5.3×10^{-2}	4.5×10^{-5}	3.5×10^{-6}	[a]slow
		35	1.3×10^{-2}	8.3×10^{-1}	9.2×10^{-3}	1.7×10^{-3}	5.7×10^{-5}
			1.1×10^{-2}	1.0	--	1.2×10^{-3}	3.2×10^{-5}
			8.7×10^{-3}	4.2×10^{-1}	7.0×10^{-3}	8.0×10^{-4}	3.6×10^{-5}
			6.3×10^{-3}	7.2×10^{-1}	4.7×10^{-3}	4.6×10^{-4}	3.3×10^{-5}
			3.2×10^{-3}	2.1×10^{-1}	--	--	--
			3.8×10^{-3}	--	2.8×10^{-3}	3.5×10^{-4}	1.5×10^{-5}
			1.3×10^{-3}	1.0×10^{-1}	7.5×10^{-4}	2.1×10^{-4}	3.2×10^{-6}
		45	1.3×10^{-2}	[b]fast	[b]fast	2.3×10^{-3}	1.6×10^{-4}
			1.1×10^{-2}	[b]fast	[b]fast	1.8×10^{-3}	8.0×10^{-5}
			8.7×10^{-3}	[b]fast	[b]fast	1.4×10^{-3}	9.0×10^{-5}
			6.3×10^{-3}	[b]fast	[b]fast	9.8×10^{-4}	7.6×10^{-5}
			3.8×10^{-3}	[b]fast	[b]fast	5.4×10^{-4}	4.3×10^{-5}
			1.3×10^{-3}	[b]fast	[b]fast	2.6×10^{-4}	2.0×10^{-5}
		55	1.3×10^{-2}	[b]fast	[b]fast	6.0×10^{-3}	2.9×10^{-4}
			1.1×10^{-2}	[b]fast	[b]fast	4.0×10^{-3}	2.6×10^{-4}
			8.7×10^{-3}	[b]fast	[b]fast	3.2×10^{-3}	2.3×10^{-4}
			6.3×10^{-3}	[b]fast	[b]fast	2.5×10^{-3}	1.7×10^{-4}
			3.8×10^{-3}	[b]fast	[b]fast	1.7×10^{-3}	1.1×10^{-4}
			1.3×10^{-3}	[b]fast	[b]fast	5.5×10^{-4}	5.0×10^{-5}
$\{Na(OH_2)\}^+$	1.0 M $HClO_4$	15	1.3×10^{-2}	5.6×10^{-2}	9.4×10^{-3}	1.0×10^{-3}	1.0×10^{-4}
			1.1×10^{-2}	4.8×10^{-2}	6.7×10^{-3}	7.3×10^{-4}	6.8×10^{-5}
			8.7×10^{-3}	4.1×10^{-2}	7.9×10^{-3}	8.8×10^{-4}	8.5×10^{-5}
			6.3×10^{-3}	2.7×10^{-2}	5.7×10^{-3}	4.9×10^{-4}	6.5×10^{-5}
			3.8×10^{-3}	1.4×10^{-2}	2.5×10^{-3}	7.0×10^{-5}	1.5×10^{-5}
			1.3×10^{-3}	1.0×10^{-2}	4.2×10^{-4}	9.0×10^{-5}	6.8×10^{-6}
		25	1.3×10^{-2}	9.9×10^{-2}	2.3×10^{-2}	4.4×10^{-3}	4.8×10^{-4}
			1.1×10^{-2}	9.2×10^{-2}	1.7×10^{-2}	3.1×10^{-3}	2.7×10^{-4}
			8.7×10^{-3}	7.1×10^{-2}	1.6×10^{-2}	2.1×10^{-3}	2.6×10^{-4}
			6.3×10^{-3}	6.0×10^{-2}	1.4×10^{-2}	1.9×10^{-3}	1.9×10^{-4}
			3.8×10^{-3}	4.3×10^{-2}	7.9×10^{-3}	8.6×10^{-4}	7.1×10^{-5}
			1.3×10^{-3}	2.5×10^{-2}	3.1×10^{-3}	5.0×10^{-4}	3.0×10^{-5}
		35	1.3×10^{-2}	2.5×10^{-1}	[b]fast	6.8×10^{-3}	4.3×10^{-4}
			1.1×10^{-2}	--	[b]fast	5.3×10^{-3}	3.7×10^{-4}
			8.7×10^{-3}	1.9×10^{-1}	[b]fast	5.5×10^{-3}	3.4×10^{-4}

			6.3×10^{-3}	9.7×10^{-2}	1.8×10^{-2}	3.1×10^{-3}	2.7×10^{-4}
			3.8×10^{-3}	9.0×10^{-2}	1.2×10^{-2}	2.3×10^{-3}	2.1×10^{-4}
			1.3×10^{-3}	[b]fast	5.2×10^{-3}	1.1×10^{-3}	1.2×10^{-4}
**REDUCTION**							
$\{Na(OH_2)\}^+$	Medium	*T* /ºC	$[OH^-]$ /M	k_{obs1} /s^{-1}	k_{obs2} /s^{-1}	k_{obs3} /s^{-1}	
		35	1.0×10^{-6}	1.2×10^{-4}	6.2×10^{-6}	--	
			2.0×10^{-6}	2.5×10^{-4}	1.6×10^{-5}	2.7×10^{-6}	
			4.0×10^{-6}	3.7×10^{-4}	4.3×10^{-5}	7.5×10^{-6}	
			6.3×10^{-6}	4.6×10^{-4}	6.8×10^{-5}	1.4×10^{-5}	
			1.3×10^{-5}	1.3×10^{-3}	1.6×10^{-4}	1.5×10^{-5}	
		45	2.0×10^{-6}	4.5×10^{-4}	3.8×10^{-5}	7.0×10^{-6}	
			4.0×10^{-6}	1.3×10^{-3}	2.8×10^{-5}	--	
	0.1 M borax		6.3×10^{-6}	2.8×10^{-3}	1.7×10^{-4}	2.5×10^{-5}	
	0.9 M $NaClO_4$		1.3×10^{-5}	3.3×10^{-3}	2.2×10^{-4}	2.5×10^{-5}	
			4.0×10^{-5}		1.0×10^{-3}	5.0×10^{-3}	
		55	1.0×10^{-6}	2.6×10^{-4}	2.0×10^{-5}	--	
			2.0×10^{-6}	--	--	--	
			4.0×10^{-6}	1.6×10^{-3}	1.0×10^{-4}	2.8×10^{-5}	
			6.3×10^{-6}	--	3.6×10^{-4}	4.9×10^{-5}	
			1.3×10^{-5}	--	1.1×10^{-3}	5.5×10^{-5}	

[a]*slow = too slow to measure under these conditions;* [b]*fast = too fast to measure under these conditions; -- = not determined*

References:

1. Gallen, A.; Jover, J.; Ferrer, M.; Martínez, M., Building a molecular PrussianBlueAnalogue FeII/CoIII cube around a Cs+ ion; a preferred, tight, robust, water soluble, and kinetically inert molecular encapsulator. *Inorganica Chimica Acta* **2023,** *545*, 121282.
2. Gonzálvez, M. A.; Bernhardt, P. V.; Font-Bardia, M.; Gallen, A.; Jover, J.; Ferrer, M.; Martínez, M., Molecular Approach to Alkali-Metal Encapsulation by a Prussian Blue Analogue FeII/CoIII Cube in Aqueous Solution: A Kineticomechanistic Exchange Study. *Inorganic Chemistry* **2021,** *60*, 18407-18422.
3. Gonzálvez, M. A.; Gallen, A.; Ferrer, M.; Martínez, M., Self-Assembly and Properties of a Discrete Water-Soluble Prussian Blue Analogue Fe^{II}/Co^{III} Cube: Confinement of a Water Molecule in Aqueous Solution. *Inorganic Chemistry* **2020,** *59*, 1582-1587.
4. Wilkins, R. G., *Kinetics and Mechanisms of Reactions of Transition Metal Complexes*. VCH: Weinheim/New York, 1991.
5. Alcázar, L.; Bernhardt, P. V.; Ferrer, M.; Font-Bardia, M.; Gallen, A.; Jover, J.; Martínez, M.; Peters, J.; Zerk, T. J., Kineticomechanistic Study of the Redox pH Cycling Processes Occurring on a Robust Water-Soluble Cyanido-Bridged Mixed-Valence $\{Co^{III}/Fe^{II}\}_2$ Square. *Inorganic Chemistry* **2018,** *57*, 8465-8475.
6. Bernhardt, P. V.; Bozoglián, F.; Macpherson, B. P.; Martínez, M.; González, G.; Sienra, B., Discrete cyanide bridged mixed-valence Co/Fe complexes: outer sphere redox behaviour. *European Journal of Inorganic Chemistry* **2003**, 2512-2518.
7. Levine, I. N., *Physical Chemistry*. MacGrawHill: 1994.
